# Experiments with the Turkish Bath

**Published:** 1877-11

**Authors:** 


### EXPERIMENTS WITH THE TURKISH BATH.

Some interesting observations were related at the last meeting of the British Medical Association, by William James Fleming, M.B. (Glasgow). These experiments were performed by the author upon himself, and consisted of observations on the effect of the Turkish bath at temperatures of from 130° Fahr., to 170° Fahr., upon the weight, temperature, pulse, respiration, and secretions. The results showed that immersion of the body in hot dry air produced loss of weight to an extent considerably greater than normal, amounting, on the average, to a rate of above forty ounces an hour. This was accompanied by an increase in the temperature of the body and a rise in the pulse rate, with at first a fall and then a rise in the rapidity of respiration. The amount of solids secreted by the kidneys was increased, and coincidently the amount of urea. The sweet contained a quantity of solid matter in solution, and among other things a considerable amount of urea. The most important effect of the bath was the stimulation of the emunctory action of the skin. By this means the tissues could, as it were, be washed by passing water through them from within out. The increased temperature and pulse-rate pointed to the necessity of caution in the use of the bath when the circulatory system was diseased.

